# Therapeutic Advantages of Dual Targeting of PPAR-δ and PPAR-γ in an Experimental Model of Sporadic Alzheimer’s Disease

**DOI:** 10.13188/2376-922X.1000025

**Published:** 2018-05-21

**Authors:** D Reich, G Gallucci, M Tong, SM de la Monte

**Affiliations:** 1 Brandeis University, Waltham University, USA; 2 Department of Medicine, University of Brown University, USA; 3 Departments of Neurology, University of Brown University, USA

**Keywords:** Alzheimer’s disease, PPAR agonists, Neurodegeneration, Intracerebral streptozotocin, PPAR-δ, PPAR-γ, Mitochondria, Brain slice culture

## Abstract

**Background:**

Alzheimer’s disease (AD) is associated with progressive impairments in brain responsiveness to insulin and insulin-like growth factor (IGF). Although deficiencies in brain insulin and IGF could be ameliorated with trophic factors such as insulin, impairments in receptor expression, binding, and tyrosine kinase activation require alternative strategies. Peroxisome proliferator-activated receptor (PPAR) agonists target genes downstream of insulin/IGF stimulation. Furthermore, their anti-oxidant and anti-inflammatory effects address other pathologies contributing to neurodegeneration.

**Objectives:**

The goal of this research was to examine effects of dual delivery of L165, 041 (PPAR-δ) and F-L-Leu (PPAR-γ) agonists for remediating in the early stages of neurodegeneration.

**Model:**

Experiments were conducted using frontal lobe slice cultures from an intracerebral Streptozotocin (i.c. STZ) rat model of AD.

**Results:**

PPAR-δ+ PPAR-γ agonist treatments increased indices of neuronal and myelin maturation, and mitochondrial proliferation and function, and decreased neuroinflammation, AβPP-Aβ, neurotoxicity, ubiquitin, and nitrosative stress, but failed to restore choline acetyl transferase expression and adversely increased HNE(lipid peroxidation) and acetylcholinesterase, which would have further increased stress and reduced cholinergic function in the STZ brain cultures.

**Conclusion:**

PPAR-δ + PPAR-γ agonist treatments have substantial positive early therapeutic targeting effects on AD-associated molecular and biochemical brain pathologies. However, additional or alternative strategies may be needed to optimize disease remediation during the initial phases of treatment.

## Introduction

Impairments in brain insulin and insulin-like growth factor (IGF) signaling are core abnormalities in the pathogenesis of Alzheimer’s disease (AD) such that the molecular and biochemical consequences closely resemble the effects of both Type 1 and Type 2 diabetes mellitus [1,2-5], including deficits glucose utilization [6-8]. The integral relationships between insulin/IGF trophic factor deficiencies and receptor resistances and neurodegeneration are rooted in the critical roles brain insulin and IGF networks have in modulating neuronal plasticity, cell survival, myelin maintenance, cellular homeostasis, neuroinflammation, oxidative, nitrosative, and endoplasmic reticulum (ER) stress, and mitochondrial function [9]. Furthermore, deficits in insulin and IGF signaling also adversely affect neuronal cytoskeleton integrity, glial-vascular functions, neuritic sprouting, and synaptic plasticity since their networks cross-talk with Notch and Wnt pathways [10,11].

Glucose is the dominant fuel for brain metabolism, and its uptake and utilization are regulated by insulin. Reductions in brain glucose metabolism develop very early in AD, including in pre-symptomatic periods [12-15], and decline with progression of disease [6-8]. Mechanistically, these responses are attributable to disease stage-associated reductions in brain insulin and IGF-1 levels, and ability to respond to insulin or IGF-1 stimulation due to decreased receptor expression, ligand binding, and receptor tyrosine kinase activation, as demonstrated in human postmortem studies [5,16,17]. In addition, downstream signaling through insulin receptor substrate (IRS) proteins, phosphatidylinositol-3-kinase (PI3K), and AKT is impaired, favoring activation of glycogen synthase kinase-3β (GSK-3β) [5,16,17]. Reduced brain levels of insulin/IGF ligands, particularly in the early stages of AD [16], may be amenable to treatment. Correspondingly, in limited clinical trials, intranasal insulin administration was shown to improve memory and executive function in participants diagnosed with early or moderate AD [18-22]. An additional strategy could be to administer in cretins to stimulate endogenous production or secretion of insulin or drive downstream physiological responses with incretin receptor agonists [23]. Although one potential drawback is that oral formulations of incretin sand incretin receptor agonists have not been developed, several long-lasting and effective injectable preparations have been approved by the U.S. Food and Drug Administration [24,25]. A third approach that would be feasible in all phases of neurodegeneration is to use insulin sensitizers, in particular, peroxisome proliferator-activated receptor (PPAR) agonists, which function at the nuclear level, bypassing the need for surface receptor binding to alter insulin pathway responses [26-33].

PPARs are nuclear hormone receptors that function as transcription factors [31,34]. PPAR-α, PPAR-β/δ, and PPAR-γ are the three subtypes expressed throughout the body. In the brain, PPAR-β/δ is most abundant, followed by PPAR-γ and then PPAR-α [28,33]. PPARs regulate gene expression by heterodimerizing with retinoid X receptors [31]. The resulting complex binds to promoter sequences of target genes [31,34], including those mediatingenergy metabolism, cell growth, differentiation, inflammation and oxidative stress [26,27,32,33,35]. Correspondingly, PPAR agonist treatments of insulin resistance states increase positive signaling through IRS and PI3K-AKT, and reduce injury-associated signaling through GSK-3β [36-39]. In the intracerebral (i.e.) Streptozotocin (STZ) model of sporadic AD and the 5XFAD model of genetic AD [3,28,40], treatment with a PPAR-δ agonist prevented or reduced deficits in spatial learning and memory, neurodegeneration, and brain levels of amyloid-β peptide cleavage product of the amyloid precursor protein (Aβ), phospho-tau (pTau), and oxidative stress [28].

The fundamental importance of PPAR-δ signaling in relation to brain structure and function is highlighted by the increased levels of pTau, the pro-inflammatory cytokine interleukin-6 (IL-6), cyclin-dependent kinase 5 (Cdk5), beta-site amyloid precursor protein cleaving enzyme 1(beta-secretase 1; BACE1) activity, and receptor for advanced glycation end-products (RAGE) in brains of PPAR-δ null mice [38]. Essentially, a broad array of AD-associated pathologies has been linked to impaired signaling through insulin/IGF pathways via PPAR-δ. Mechanistically, in PPAR-δ gene depleted mice, aberrant tau phosphorylation could have been due to increased Cdk-5 activation, neuroinflammation, impaired insulin and IGF signaling, or oxidative stress [1,41,42]. Increased BACE1 activity could be attributed to insulin resistance phenotypic effects of depleting PPAR-δ since insulin resistance increases BACE1 activity and metformin treatment of other AD models reduces BACE1 and Aβ generation [43]. Advanced glycation end-products (AGE) increase in insulin resistant disease states, corresponding with the effects of PPAR-δ depletion. However, AGEs contribute to AD by promoting oxidative stress, inflammation, and insulin resistance [44], and increased expression of RAGE in AD enhances Aβ neurotoxicity, possibly via its increased transport across the blood-brain barrier and attendant accumulation in the brain [45-47]. Overall, the findings in the PPAR-δ null mouse model strongly support the hypothesis that AD neurodegeneration can result from chronic impairments in insulin signaling through PPAR-δ accompanied by progressive increases in AGE, RAGE activity, neuroinflammation, oxidative stress, and Aβ accumulations [45,47].

PPAR-γ also mediates insulin’s actions in the brain as demonstrated by the neuroprotective responses observed in humans with AD [48], and in the Tg2576 and 3xTg-AD genetic mouse models of AD [49,50]. In addition, diabetics treated with the PPAR-γ agonist, rosiglitazone, exhibited better stabilization of long-term cognitive function compared with subjects treated with metformin [51]. Pioglitazone, another PPAR-γ agonist, was shown to restore deficits in synaptic transmission, enhance long-term potentiation, restore dendritic spine densities and adaptive plasticity responses damaged by Aβ42 [50], and enhance brain oxygen consumption [48]. However, it is doubtful that treatment with PPAR-γ agonists should be the single or dominant form of insulin sensitizer therapy in AD because: 1) the brain expresses predominantly PPAR-δ followed by PPAR-γ, which have overlapping but non-identical functions [29]; and 2) the L165,041 PPAR-δ agonist was shown to be superior to F-L-Leu PPAR-γ agonist in rescuing neurodegeneration and deficits in spatial learning and memory in the i.c. STZ model of sporadic AD [28]. Of note is that the GW7647 PPAR-α agonist was therapeutically ineffective in the i.c. STZ model. Altogether, the findings suggest that both PPAR-δ and PPAR-γ agonists are needed for effective therapeutic targeting in AD. The goal of these experiments was to determine if simultaneous targeting of PPAR-δ and PPAR-γ in the brain would be effective in remediating the i.c. STZ model of neurodegeneration.

## Methods

Frontal lobe slice cultures: Postnatal day 3 (P3) Long Evans rat pups were used in these experiments because adult brains are not suitable for long-term slice cultures. Detailed methods were described previously [28,52]. In brief, the pups were administered i.c. STZ (0.5 mg) or vehicle (saline), and 24 h later, their frontal lobes were harvested for slice culture. After washing twice in Ca^2+^/Mg^2+^-free Hank’s balanced salt solution (HBSS) (4° C), the frontal lobes were positioned onto the plastic mounting tray of a McIlwain Tissue Chopper (Ted Pella, Inc., Redding, CA) and sliced at a thickness of 250 μm. The slices were separated under a dissecting microscope and transferred to culture wells (12-well plates; 3 slices/well) containing 300 μL Dulbecco’s Modified Eagle’s medium (DMEM) supplemented with 10% fetal bovine serum (FBS), 4 mM glutamine, 25 mM KC1, 10 mM non-essential amino acids, and 120 U/mL each of penicillin and streptomycin. Cultures were maintained at 37° C in a standard C0_2_ incubator. The cultures were treated with PPAR-δ (L165,041;10 pM) + PPAR-γ (F-L-Leu; 20 μM) agonists, or vehicle by daily addition to fresh media. The cultures were maintained for 72 hours, after which the supernatants were harvested for cytotoxicity assays and the tissue slices were cryo-preserved at −80° C.

### Cytotoxicity assay

Cytotoxicity was measured with the Vybrant Cytotoxicity Assay Kit (Molecular Probes, Eugene, OR) according the manufacturer’s protocol. In brief, 50 μL of culture supernatant were transferred to a white Opti Plate (PerkinElmer, Waltham, MA) and incubated (30 minutes at 37° C) with reaction mixture containing 4 mM resazurin. Fluorescence intensity measured in a SpectraMax M5 microplate reader (Molecular Devices Corp., Sunnyvale, CA; Ex/Em: 530/590 nm) was normalized to protein concentration in the tissue samples.

### Duplex Enzyme-Linked Immunosorbent Assay (ELISA)

Fresh frozen slice culture tissues were homogenized in NP-40 lysis buffer supplemented with protease and phosphatase inhibitors [36]. Protein concentrations were determined with the bi cinchoninic acid (BCA) assay (Pierce, Rockford, IL). Immunoreactivity was measured in 4 replicate 100 ng protein samples by direct binding ELISA [36], and protein loading was subsequently quantified by measuring immune reactivity to large acidic ribosomal protein (RPLPO) [36]. Primary antibodies were diluted to 0.1-0.5 pg/ml, and their binding was detected with horseradish peroxidase (HRP)-conjugated secondary antibody (1:10000; Pierce, Rockford, IL) and Amplex Ultra Red soluble fluorophore (Molecular Probes, Eugene, OR). Amplex Red fluorescence fluorescent light units (FLU) were measured in a SpectraMax M5 (Ex 530/Em 590). Subsequently, the samples were incubated with biotin-conjugated polyclonal antibodies to RPLPO, and immunoreactivity was detected with streptavidin-conjugated alkaline phosphatase (1:1000; Vector, Burlingame, CA) and the 4-Methylumbelliferyl phosphate (4-MUP) fluorophore (Molecular Probes, Eugene, OR) (Ex360/Em450). Binding specificity was assessed with negative control incubations in which the primary or secondary antibody was omitted. The calculated ratios of specific protein/RPLPO fluorescence were used for inter-group statistical comparisons.

### Statistics

Box plots depict the means (horizontal bars), 95% confidence interval limits (upper and lower boundaries of the boxes), and range (stems). Inter-group comparisons were made using two-way analysis of variance (ANOVA) with the Holm-Sidak multiple comparisons post hoc test (Graph Pad Prism 7, San Diego, CA). Significant posttest differences (p<0.05) are shown in the graphs and tables.

### Sources of reagents

Antibodies to tau (ab64193), S396 (ab 109390) and T205 (ab4841) phospho-tau, ubiquitin (UBI-1 ab7780), myelin-associated glycoprotein (MAG-1; ab89780), glial fibrillary acidic protein (GFAP; ab7260), 3-nitrotyrosine (NTyR; ab61392), Hu neuronal marker (ab78467), Complex IV (abl4744), ATP synthase (abl81243), 8-hydroxyguanosine (8-OHdG; abl0802), common leukocyte antigen (CD45; abl0558), and 4-hydroxynonenal (HNE; ab46545), and the EDH cytotoxicity assay were purchased from Abeam (Cambridge, MA). Rabbit polyclonal antibody to RPEPO (RPE23 16086-1-AP) was purchased from Proteintech Inc (Chicago, IL). Amplex Ultra Red soluble HRP fluorophore and 4-MUP alkaline phosphatase substrate were purchased from Invitrogen (Carlsbad, CA). The L165, 041 PPAR-δ agonist and F-L-Leu PPAR-γ agonist were purchased from Cal Biochem (San Diego, CA).

## Results

### STZ and PPAR agonists effects on neuroglial protein expression

**Figure 1:**
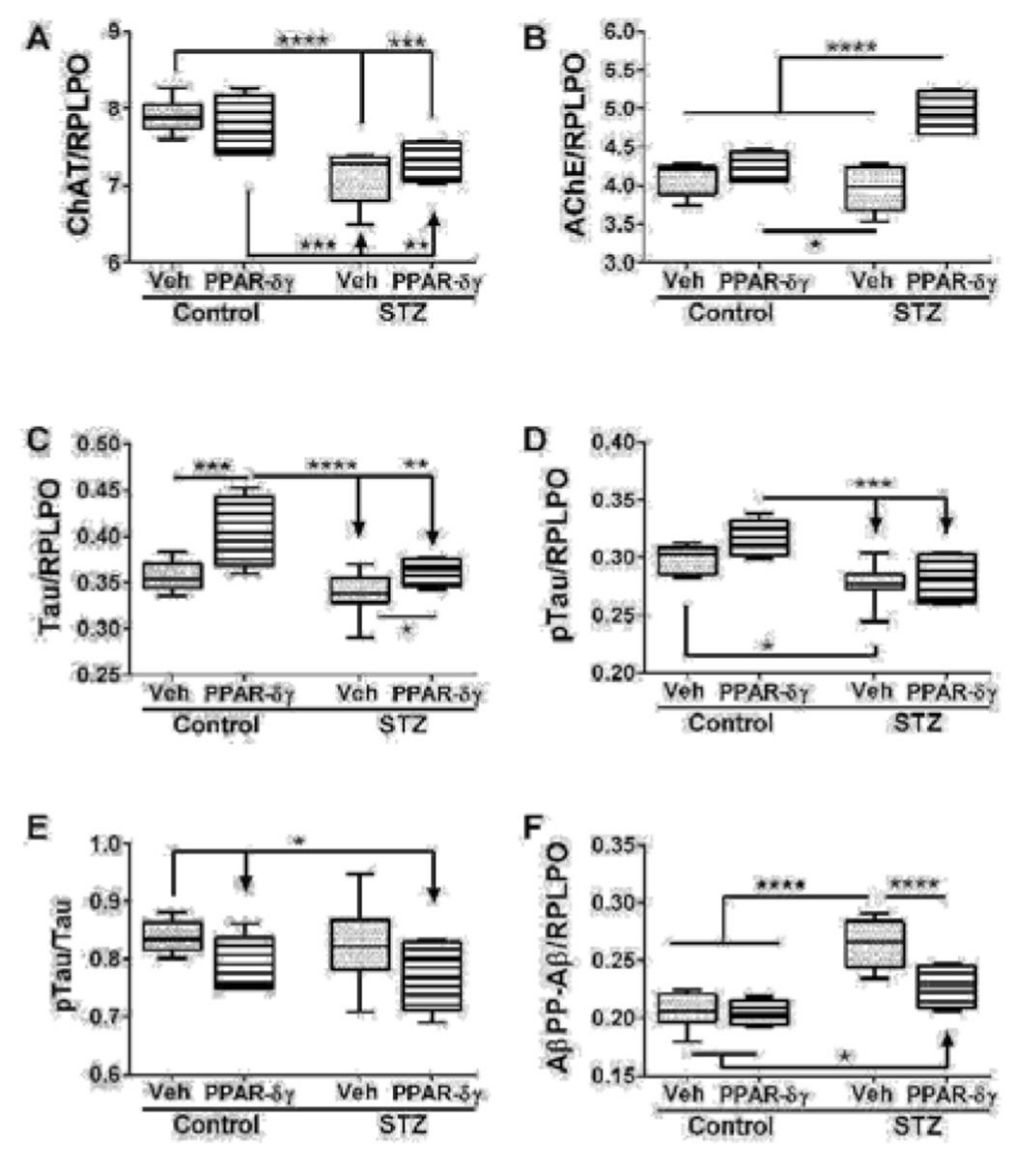
Effects of PPAR agonist treatments on i.e. STZ-aIterations in neuroglial and inflammatory markers. Postnatal Day 3 (P3) Long Evans rat pups were treated with i.e. STZ or vehicle. Frontal lobe slice cultures generated 24 hours later were maintained for 72 hours and treated daily with L165,041 (PPAR-δ) + F-L-Leu (PPAR-γ) agonists or vehicle. Protein homogenates were used to measure (A) Hu (neuronal marker), (B) common leukocyte antigen-microglia (CD45), (C) myelin-associated glycoprotein-1 (MAG-1), and (D) glial fibrillary acidic protein (GFAP) immunoreactivity with duplex ELISAs. Results were normalized to RPLPO. Data were analyzed by two-way ANOVA (Table 1). Significant post hoc test results are displayed.

Duplex ELISAs were used to measure immunoreactivity to Hu (an immature neuronal marker), CD45 (microglia), MAG-1 (mature myelin protein), and GFAP (astrocyte marker) (Table 1 and Figure 1). The i.c. STZ treatments had significant effects on Hu, CD45, and MAG-1, but not GFAP expression. The PPAR agonists significantly altered expression of Hu and CD45, and had a trend effect on GFAP, but no significant effect on MAG-1. Significant STZ × PPAR agonist interactive effects only occurred with respect to MAG-1 (Table 1). Post hoc tests demonstrated that Hu expression significantly declined with PPAR-δ + PPAR-γ treatments of both control and STZ samples, indicating positive effects on neuronal maturation (Figure 1A). The expression levels of the CD45 microglial marker were significantly elevated in vehicle-treated STZ versus all other groups (Figure 1B). Treatment of control and STZ cultures with PPAR-δ + PPAR-γ significantly reduced CD45 expression relative to respective vehicle, corresponding with the anti-inflammatory effects of PPAR agonists. MAG-1 expression was significantly reduced in vehicle-treated STZ cultures relative to both control groups (Figure 1C). PPAR-δ + PPAR-γ treatments increased MAG-1 in the STZ cultures, but the levels were still lower than vehicle-treated control (Figure 1C). Finally, GFAP expression was similar across all groups, except that PPAR-δ + PPAR-γ treatment of the STZ cultures increased the levels above those in STZ + Vehicle but not the control cultures (Figure 1D).

### Indices of neurodegeneration

Duplex EFISAs were used to measure choline acetyl transferase (ChAT), acetyl cholinesterase (AChE), total Tau, pTau, and Aβ. ChAT, AChE, pTau, and AβPP-Aβ expressions were significantly modulated by i.c. STZ. PPAR agonist treatments significantly impacted AChE, Tau, and AβPP-Aβ expression (Table 1). Interactive effects of STZ and PPAR agonists were detected with respect to AChE and AβPP-Aβ expression (Table 1).

**Figure 2:**
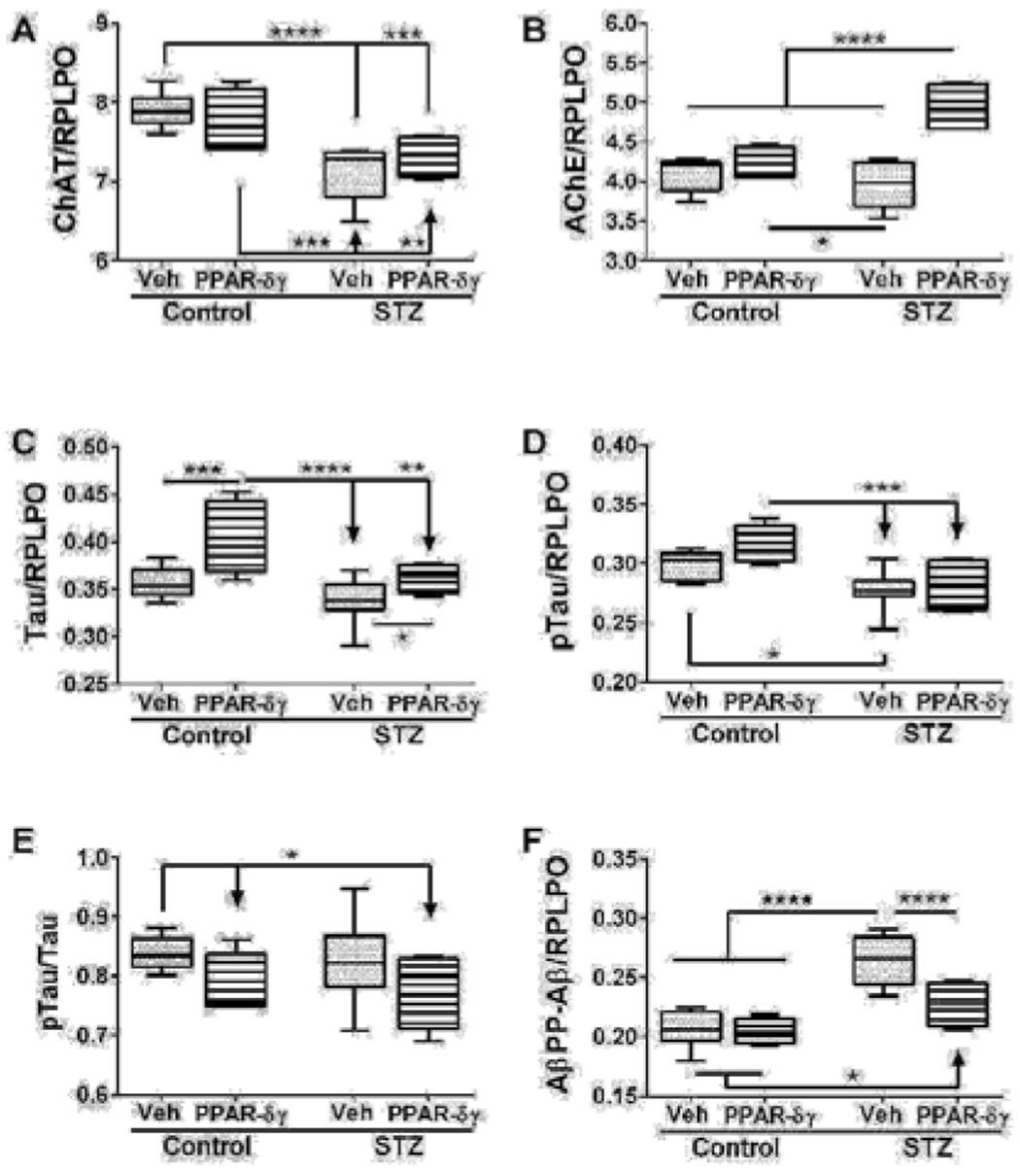
PPAR agonist rescue of i.c. STZ induced AD-type neurodegeneration. Frontal lobe slice cultures from i.c. STZ- and vehicle-treated control rat pups were treated for 72 hours with PPAR-δ + PPAR-γ agonists or vehicle (See Legend to Figure 1). Duplex ELISAs measured (A) choline acetyltransferase (ChAT), (B) acetylcholinesterase (AChE), (C) Tau, (D) pTau, (E) pTau/Tau calculated mean ratios, and (F) amyloid-beta peptide (1-42) of amyloid beta precursor protein (AβPP-Aβ) with results normalized to RPLPO. Data were analyzed by two-way ANOVA (Table 1). Significant post hoc test results are displayed.

Corresponding with the 2-way ANOVA results, post hoc tests demonstrated significant STZ-mediated suppression of ChAT and no significant remediation by the PPAR-δ + PPAR-γ treatments (Figure 2A). The dominant effect observed with respect to AChE was that the mean levels were highest in the STZ + PPAR agonist treated group relative to all others (Figure 2B). In addition, STZ (+vehicle) reduced AChE expression relative to control + PPAR agonist. In control cultures, PPAR-δ + PPAR-γ treatments significantly up-regulated Tau expression above the other 3 groups. In addition, PPAR agonists significantly increased Tau in the STZ cultures relative to the control (Figure 2C). The mean levels of pTau were highest in control cultures treated with PPAR-δ + PPAR-γ and lowest in STZ + vehicle cultures (Figure 2D) corresponding with the effects of PPAR agonists and STZ on Tau protein expression. However, the calculated mean ratios of pTau/Tau were significantly reduced by PPAR-δ + PPAR-γ treatments relative to control (Figure 2E). STZ significantly increased AβPP-Aβ immune reactivity relative to all other groups (Figure 2F). Although treatment with PPAR-δ + PPAR-γ agonists significantly reduced AβPP-Aβ levels in the STZ cultures, the resulting levels were still higher than control (Figure 2F).

### PPAR agonists effects on cytotoxicity and mitochondrial dysfunction

**Figure 3:**
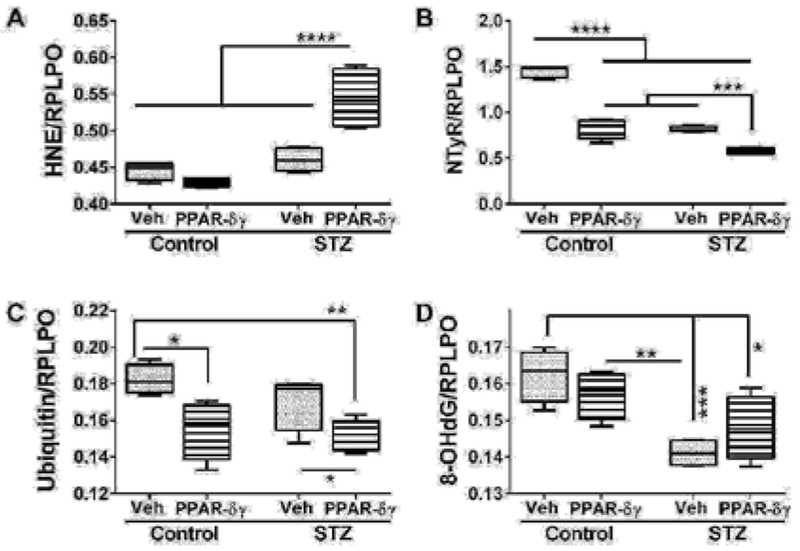
PPAR agonist inhibition of cytotoxicity and modulation of mitochondrial mass and function in the in i.c. STZ model of AD neurodegeneration. Long Evans rat pups were treated with i.c. STZ or vehicle. Frontal lobe slice cultures were maintained for 72 hours with daily PPAR-δ + PPAR-γ agonists or vehicle treatments. At the conclusion of the experiment, (A) LDH release was measured in culture supernatants and (B) mitochondrial mass was measured using the MitoTracker Green (MTG) fluorescence assay. LDH and MTG were normalized to Hoechst’s H33342 fluorescence, corresponding to cell number. Immunoreactivity to (C) cytochrome oxidase (COX)-Complex IV and (D) ATP Synthase (ATP SYN)-Complex V were measured by duplex ELISA with results normalized to RPLPO. Data were analyzed by two-way ANOVA (Table 1). Significant post hoc test results are displayed.

The FDH cytotoxicity assay results demonstrated significant interactive effects between STZ and PPAR agonists, and a trend effects for PPAR agonists (Table 1). Post hoc tests demonstrated significantly higher levels of LDH release in the STZ + vehicle treated cultures and normalization of LDH release, i.e. significantly reduced STZ-induced cytotoxicity by PPAR agonist treatments (Figure 3A). STZ had significant effects on cytochrome C oxidase (COX; Complex IV) and ATP synthase (Complex V), and trend effects on mitochondrial mass (MitoTracker Green; MTG), whereas PPAR agonist treatments significantly altered COX, ATP Syn, and MTG levels. There were no significant STZ × PPAR agonist effects on COX, ATP Syn or MTG. Post hoc tests demonstrated that the PPAR agonist treatments significantly and similarly increased MTG levels, i.e. mitochondrial mass/proliferation in both control and STZ cultures (Figure 3B). PPAR-δ + PPAR-γ agonists increased COX expression in STZ cultures such that the mean levels were significantly higher than in the other three groups, whereas the stimulatory effects in control cultures were not statistically significant (Figure 3C). The main response observed with respect to ATP synthase was that the PPAR-δ + PPAR-γ agonists significantly reduced its expression in control cultures relative to all other groups (Figure 3D). No significant inhibitory effect occurred due to STZ.

### PPAR agonist effects on indices of cellular stress

**Figure 4:**
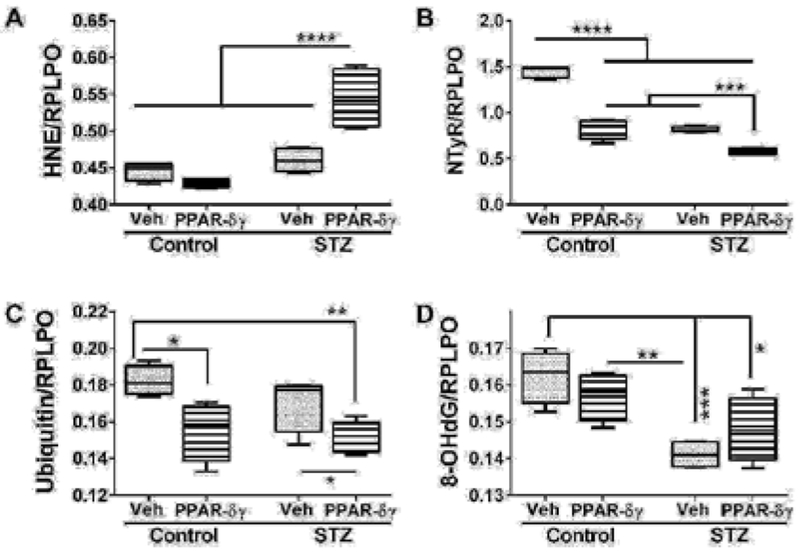
PPAR agonist treatment effects on STZ-induced indices of oxidative stress. Frontal lobe slice cultures from i.c. STZ or vehicle injected rat pups were maintained for 72 hours and treated daily with PPAR-δ + PPAR-γ agonists or vehicle. I mm u noreactivity to (A) 4-hydroxy-nonenal (HNE), (B) Nitrotyrosine (NTyr), (C) ubiquitin, and (D) 8-hydroxydeoxyguanosine (8-OHdG) was by measured by duplex ELISA with results normalized to RPLPO. Data were analyzed by two-way ANOVA (Table 1). Significant post hoc test results are displayed.

Duplex ELISAs measured immunoreactivity to 4-hydroxynonenal (HNE), nitrotyrosine (NTyR), ubiquitin, and 8-hydroxydeoxyguanine (8-OHdG). Both HNE and NTyr were significantly modulated by STZ and PPAR agonist treatments and their interactive effects (Table 1). Ubiquitin expression was significantly altered by PPAR agonist treatments, and 8-OHdG was significantly altered by STZ (Table 1). Post hoc tests demonstrated that HNE was significantly elevated in STZ cultures treated with PPAR-δ + PPAR-γ relative to all other groups (Figure 4A). In contrast, NTyr levels were significantly higher in vehicle-treated control cultures relative to all other groups. PPAR-δ + PPAR-γ agonist treatments significantly reduced NTyR immunoreactivity relative to corresponding vehicle-treated cultures (Figure 4B). Similarly, ubiquitin immunoreactivity was significantly reduced by PPAR-δ + PPAR-γ treatments in both control and STZ cultures (Figure 4C). In control and STZ cultures, 8-OHdG immunoreactivity was not significantly altered by the PPAR agonist treatments. Instead, STZ irrespective of PPAR agonist treatments, reduced 8-OHdG levels relative to control (Figure 4D).

## Discussion

The premise of this work stemmed from human and experimental data showing that AD is associated with impairments in brain insulin and IGF signaling [1,9,53-56], due in part to reduced trophic factor levels, receptor binding, receptor tyrosine kinase activation, and downstream signaling through insulin receptor substrate, PI3 Kinase and Akt [1,5,9,16,17,55]. The adverse effects of chronically impaired brain insulin and IGF signaling are substantial because these pathways have pivotal roles in regulating neuronal growth, plasticity, survival, energy metabolism, and cytoskeletal structure, myelin maintenance, and cholinergic function [4,9,57,58]. Deficiencies in ligand expression or availability could be accommodated by exogenous insulin or IGF-1 delivery such as via the intranasal route [18-20], or perhaps by treatment with incretins, which stimulate endogenous production or secretion of insulin and IGF-1 or incretin receptor agonists [1, 23]. However, as AD progresses, the fundamental abnormality that could eventually limit therapeutic efficacy of trophic factor administration is progressive brain insulin and IGF resistance, or reduced ability to respond to trophic factor stimulation [16]. One potential strategy shown to circumvent this barrier in experimental animals [28,36] and to some extent in humans [59,60], is to use insulin sensitizer drugs such as PPAR agonists. PPAR agonists remain attractive because these small molecules cross the blood-brain barrier, are effective for remediating neurodegeneration in ng/mL concentrations [28,36], and they modulate gene expression by targeting nuclear receptors [31,34,61], by-passing abnormalities at the cell surface. In addition, PPAR agonists target neuro-inflammatory and stress-mediated factors driving neurodegeneration [27,36,62,63].

One critical aspect of using PPAR agonists to treat brain diseases is that attention must be given to the fact that the dominant PPAR subtypes expressed in the central nervous system are delta, followed by gamma [1,40,58]. Perhaps the main reason for the limited therapeutic successes using PPAR agonists to treat neurodegeneration is that the clinical trials employed agonists that selectively bind to PPAR-γ. Although the neuroprotective effects of PPAR-δ and PPAR-γ agonists overlap, they do not share identical downstream insulin-responsive targets [29,31,32,34]. Correspondingly, in an earlier *in vivo* study, it was demonstrated that the L165, 041 PPAR-δ agonist was superior to the F-L-Leu PPAR-γ agonist for treating the i.c. STZ model of AD-type neurodegeneration [28]. Furthermore, subsequent experiments showed that T3D-959, a novel hybrid PPAR-δ/γ agonist, could also effectively remediate the i.c. STZ model of neurodegeneration with long-term *in vivo* responses that were similar to those observed here in [36,52,64,65]. Given the asymmetric expression of PPAR-δ and PPAR-γ in the brain, the dual nuclear receptor actions of T3D-959 permit activation of both PPAR-δ and PPAR-γ in the same cells and with a 15-fold higher affinity for PPAR-δ (primary target) than PPAR-γ (secondary target) [64,66]. In addition, the attractiveness of T3D-959 for treating humans with AD is bolstered by its effectiveness following once daily oral administrations [64,66, 67].

The experiments designed for the present paper assessed the effectiveness of dual treatments with L165, 041 + F-L-Leu for remediating early molecular and biochemical indices of neurodegeneration in the i.c. STZ rat model of neurodegeneration. Previous studies using the same model showed that i.c. causes neurodegeneration with brain insulin resistance accompanied by impaired signaling downstream through PI3K-Akt pathways [28,68-70]. It was interest to determine if the administration of two independent drugs with different PPAR targets could significantly reduce expression of multiple biomarkers of AD. Short-term experiments were done to better understand the early processes that lead to disease remediation. Although the precise mechanisms in which i.c. STZ causes neurodegeneration are not understood, the resulting neurobehavioral, histopathologic, molecular, metabolic, and neuroinflammatory abnormalities are shared with those in sporadic AD, making it an attractive model that can be reproduced in any standard laboratory [9,69-71].

Corresponding with previous reports, i.c. STZ significantly increased neuroinflammation, cytotoxicity, and AβPP-Aβ, and reduced expression of a mature myelin-associated protein (MAG-1) and ChAT [52,65]. However, we also observed significant reductions in 3-Nitrotyrosine and 8-OHdG which are discordant with previous observations [52,65]. One potential explanation for these differences is that elevated levels of NTyR and 8-OHdG, as occur in AD, represent chronic rather than acute (early) effects of neurodegeneration.

PPAR agonist treatments reduced Hu (immature neuronal marker) and CD45 (microglial marker) PPAR-δ + PPAR-γ agonist treatments were to enhance neuronal maturation, mature myelin protein expression, and astrocyte function, and reduce neuroinflammation. It is noteworthy that Hu and CD45 expression were also significantly reduced by PPAR-δ + PPAR-γ agonist treatments of control cultures, reflecting the importance of their signaling networks for maintenance of normal brain functions. The PPAR-δ + PPAR-γ agonist associated increase in GFAP expression in STZ cultures although significant relative to vehicle, was still within the range of control cultures. Potential positive effects of modestly increased but nonetheless normal levels of GFAP include increased integrity of the blood-brain barrier [72], and support of neuronal conductivity and myelination [73].

PPAR-δ + PPAR-γ failed to reverse STZ-mediated ChAT inhibition, despite *in vivo* evidence to the contrary, suggesting that stimulation of ChAT represents a long-term *in vivo* response to PPAR agonist treatments. AChE was significantly increased in STZ cultures treated with the PPAR-δ + PPAR-γ agonists. This response may have been undesirable in light of the extant cholinergic deficit since increased AChE activity could worsen cognitive function and potentially challenge *in vivo* therapeutic measures focused on increasing ChAT with cholinesterase inhibitors.

Tau is an important neuronal cytoskeletal protein that is regulated by insulin and IGF-1.PPAR-δ + PPAR-γ increased Tau expression in both control and STZ cultures and therefore served to fortify the neuronal cytoskeletal network, although the responses were more robust in control cultures. In contrast, pTau expression was not significantly increased relative to vehicle by PPAR-δ + PPAR-γ treatments of either control or STZ cultures, accounting for the modest declines in pTau/Tau, i.e. reduced relative phosphorylation, opposing potential trends toward Tau hyperphosphorylation.

Finally, PPAR-δ + PPAR-γ significantly reduced AβPP-Aβ levels in the STZ cultures. Although the levels were not normalized after short-term treatments, the trend is consistent with previous longterm *in vivo* experimental responses to T3D-959 [36,52], PPAR-δ agonists [28,40] and PPAR-γ agonists [50]. The findings suggest that PPAR-δ + PPAR-γ agonist treatments could effectively inhibit AβPP-Aβ accumulation and neurotoxicity in early-stage AD.

Mechanistically, the neuroprotective actions of PPAR-δ + PPAR-γ included significant reductions in STZ-mediated cytotoxicity (EDH release) and expansion of mitochondrial mass (MTG). PPAR-δ + PPAR-γ treatments increased MTG levels in control and STZ cultures. Increased mitochondrial mass induced by PPAR-δ + PPAR-γ was accompanied by modest increases in COX (significant for the STZ group) and reductions in ATP Synthase (significant for the control group). With only modest gains in mitochondrial function *vis-a-vis* sharply increased mitochondrial mass, it is likely that proliferated mitochondria were not optimally functional, possibly due to immediate toxic and either irreversible or slowly reversible effects of STZ.

HNE, a marker of oxidative stress and lipid peroxidation, accumulates in cells and tissues and promote DNA damage and mitochondrial dysfunction in various disease states, including neurodegeneration and diabetes mellitus [74]. Since PPAR agonists have cytoprotective and anti-oxidant effects, the significance of this specific early response is uncertain. The findings that both NTyR and ubiquitin were significantly reduced by PPAR-δ + PPAR-γ in control and STZ cultures suggest that early responses to PPAR-δ + PPAR-γ agonist treatments in sporadic AD include reductions in nitrosative stress and ubiquitination linked to neurodegeneration.

## Conclusion

In conclusion, dual PPAR-δ + PPAR-γ agonist treatments had significant early therapeutic effects on indices of neuronal (increased tau, decreased Hu) and oligodendrocyte (MAG-1) maturation, cytotoxicity, neuroinflammation (CD45), AβPP-Aβ accumulation, and oxidative/nitrosative stress in the STZ-treated cultures. In addition, PPAR-δ + PPAR-γ agonist treatments induced mitochondrial proliferation, which ultimately could help increase or stabilize mitochondrial enzymes (Complexes IV) needed to generate ATP. On the other hand, the PPAR-δ + PPAR-γ agonist treatments were ineffective in restoring ChAT and fully normalizing levels of MAG-1 and AβPP-Aβ, and they had potentially adverse effects of increasing AChE and HNE in STZ brain cultures. Altogether, the findings support the concept that simultaneous targeting of both PPAR-δ and PPAR-γ predominantly provides early neuroprotection against neurodegeneration linked to insulin and IGF resistance, but the responses are incomplete. Conceivably, PPAR-δ and PPAR-γ agonist therapeutic restoration of ChAT expression and complete normalization of mature myelin-associated protein and AβPP-Aβ expression may require longer durations of treatment. These considerations will be addressed with longer duration *in vivo* experiments in which the therapeutic responses to single versus dual PPAR-δ + PPAR-γ agonist administrations are compared. Alternatively, optimum therapeutic remediation may be achievable by targeting other aspects of cellular metabolism and stress responses, or by utilizing a drug such as T3D-959, which has hybrid dual nuclear receptor actions that permit activation of both PPAR-δ and PPAR-γ in the same cells [64,66]. In addition, an important feature of T3D-959 is that its affinity for PPAR-δ (human ED50=19 nM) is 15-times higher than PPAR-γ (human ED50=297 nM), corresponding with their proportional expression levels in the brain [64,66].

## Figures and Tables

**Table 1:** PPAR-δ + Agonist Effects in the Intracerebral Streptozotocin (STZ) Model of Sporadic Alzheimer’s Disease (2-way ANOVA results).

Protein	STZ Effect		PPAR Agonist Effect		STZ × PPAR Agonist	
	F-Ratio	P-Value	F-Ratio	P-Value	F-Ratio	P-Value
**HNE**	29.90	0.0001	8.24	0.014	18.0	0.001
**NTyr**	161.4	<0.0001	153.5	<0.0001	30.16	0.0001
**Ubiquitin**	1.68	N.S.	14.06	0.003	0.30	N.S.
**8-OHdG**	39.11	0.0008	0.038	N.S.	3.2	N.S.
**LDH**	1.1	N.S.	3,38	*0.09*	7.69	0.017
**MTG**	4.08	*0.07*	41.61	<0.0001	0.41	N.S.
**COX**	20.1	0.0003	7.02	0.02	1.31	N.S.
**ATP Syn**	9.43	0.01	16.77	0.001	1.94	N.S.
**ChAT**	33.39	<0.0001	0.06	N.S.	2.59	N.S.
**AChE**	10.62	0.004	40.33	<0.0001	22.70	<0.0001
**Tau**	0.12	N.S.	7.01	0.011	0.1	N.S.
**pTau**	21.20	<0.0001	3.14	*0.08*	0.98	N.S.
**AβPP-Aβ**	48.71	<0.0001	11.36	0.002	9.50	0.005
**Hu**	7	0.021	20.02	0.0008	0.32	N.S.
**CD45**	40.02	<0.0001	20.58	<0.0001	1.27	N.S.
**MAG-1**	14.65	0.0007	0.15	N.S.	3.95	0.012
**GFAP**	0.49	N.S.	3.63	0.07	1.44	N.S.
**H33342**	1.11	N.S.	76.16	<0.0001	1.00	N.S.
